# Behavioural development of school-aged children who live around a multi-metal sulphide mine in Guangdong province, China: a cross-sectional study

**DOI:** 10.1186/1471-2458-9-217

**Published:** 2009-07-03

**Authors:** Qing-Song Bao, Ci-Yong Lu, Hong Song, Mao Wang, Wenhua Ling, Wei-Qing Chen, Xue-Qing Deng, Yuan-Tao Hao, Shaoqi Rao

**Affiliations:** 1 Department of Food Safe Supervision, Health Inspection and Supervision Center, 205 Renmin South Road, Yixing, PR China; 2 Department of Medical Statistics and Epidemiology, School of Public Health, Sun Yat-Sen University, Guangzhou, PR China; 3 Department of Preventive Medicine, School of Public Health, Sun Yat-Sen University, Guangzhou, PR China; 4 Department of Nutrition, School of Public Health, Sun Yat-Sen University, Guangzhou, PR China

## Abstract

**Background:**

The deleterious biological effects of low-level, long-term exposure to heavy metals are well known, and children are the most susceptible population. Dabaoshan Mine in the southeast of Guangdong Province is at high risk of discharging multi-metals pollutants into a local river (Hengshihe) and the surrounding area. The present study aimed to estimate relationships between measured multi-metal exposures and the presence of behavioural problems for the school-aged children in the polluted area.

**Methods:**

A cross-sectional study was performed. Children aged 7–16 years living in three villages of the Hengshihe area with different degrees of heavy-metal pollution participated in this study. Local environmental samples (water and crops) and children's hair were collected, and concentrations of heavy metals were determined. The Child Behaviour Check-list (CBCL) was used to assess the presence of behaviour problems. General linear regression was used to analyze the contribution of hair metals to each CBCL subscale with adjustment for socio-demographic confounding factors.

**Results:**

Multiple regression analyses revealed significant effects of hair lead, cadmium and zinc levels on CBCL subscales. Log-transformed hair lead, cadmium and zinc levels accounted for an incremental of 8% to 15% variance in anxious/depressed, withdrawn, somatic complaints, social problems, thought problems, attention problems, delinquent behaviour and aggressive behaviour. The concurrent log-transformed hair lead and zinc levels were strongly associated with all subscales while the concurrent log-transformed hair cadmium was only significantly associated with withdrawn, social problems and attention problems.

**Conclusion:**

This study reveals that heavy metal exposure was associated with increased risk of behavioral problems for school-aged children.

## Background

The Dabaoshan Mine, built in 1958, is located in the southeast of Shaoguan City, Guangdong Province, and is considered as a large-scale and integrative quarrying mine, which plays an important role as a base for both nonferrous metal materials and steel industry in southern China. The Dabaoshan Mine is a multi-metal sulphide mineral deposit. The superior part of orebody is of limonite with a reservoir of 20 million tons, and the inferior part of orebody is of copper-sulphide with a reservoir of 20 million tons. Since the 1970's, the mineral separation has been incomplete in the Dabaoshan Mine, and most of the lean ore was discarded and became gradually weathered. During the operation, substantial waste water was discharged directly into the environment after leaching, ore dressing, and washing processes. Both discarded ore and waste water constitute the severe environmental pollutants to the surrounding and downstream areas.

The acid excretion water from the Dabaoshan Mine contains high content of heavy metals. Previous surveys [1,2] showed that the PH of the excretion water was 2.15; and heavy metal content in the excretion water was very high. Concentrations of cadmium, zinc and lead in the irrigation water of the nearby crop region were 16 times, 3 times and 0.5 times higher than standards as the government suggests (The Standards for Irrigation Water Quality, GB 5084-1992), respectively; and there was not any bentho in the 25 km extent from the debris dam in the mine to the polluted downstream river. In contrast, there were 36 kinds of benthos in the upstream river.

With increases of the total metal amount, convertible metal amount in soil and acidity, the soil biological availability was reduced,. A previous survey [3] found that the PH of the soil near the mine was about 4.0 and the heavy metal content in the soil of the Dabaoshan Mountains was very high, with the concentration of lead and cadmium in the soil being 44 times and 12 times higher than the government standard (The Environmental Quality Standard for Soils, GB 15618-1995), respectively. And the surface-layer soil in the coastal area of the polluted river aggregated a large amount of lead, cadmium and zinc.

Hengshihe River, as the main drainage pathway for the excretion water of Dabaoshan Mine, has been polluted since the 1970s. The river delivers significant amount of heavy metals to numerous villages. After about 30 years of exposure to these metals, some local residents in the Hengshihe area began to acquire enteron diseases, such as colon cancer. Children were the most susceptible to heavy metal contamination because they absorbed a higher percentage and excreted a lower percentage of metals than adults did. The deleterious effects of the low-level, long-term exposure to heavy metals on children, especially lead, are well known [4-7]. Behavioural disturbances, impaired mental development, and decrements in cognitive function are typical subclinical sign of intoxication in children [4-7].

Increasing attention has been paid to the pollution issues in the Hengshihe area. Ecosystem reparation is generally believed to be a reasonable way to control the pollution issues in the affected area. However, this procedure would take as long as more than 20 years, to demonstrate significant protective effects. Therefore, the health care for the current residents in the polluted area requires alternative means, which rely on our knowledge about the health status of the local residents. Up to the present, there is not a study to investigate the detrimental effects of heavy metals on children's health in the affected area.

The present study aimed to determine the environmental exposure in water, field crops and children's hair in the area and test the relationships between these exposures and behavioural problems among children aged 7–16 years old.

## Methods

### Study population

**Figure 1:**
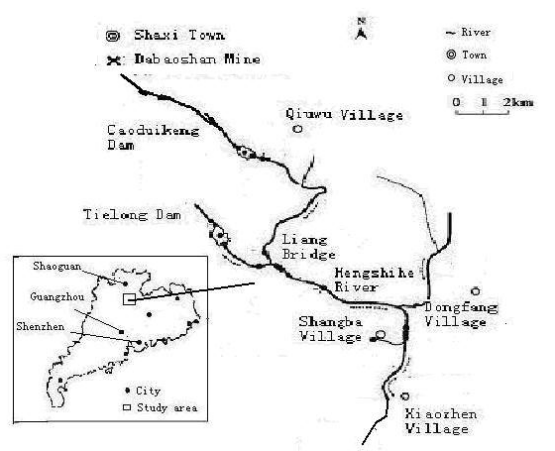
**Geographic map of Dabaoshan mine and the surrounding area**.

This cross-sectional study was conducted in three villages (Xiaozhen, Shangba, and Dongfang Village) around the downstream river of Hengshihe in Shaoguan City in August, 2006. Both drinking water and irrigation water for Xiaozhen and Shangba villages were from Hengshihe River, while the drinking water and irrigation water for Dongfang Village were from ground water. The geographic locations for the three villages are shown in Figure 1. All of the students in both elementary schools and middle schools living in these three villages, aged from 7 to 16 years, participated in the emotional and behavioural development survey. Among the total 562 eligible school-aged children, 549 (97.6%) completed the survey. The study received approval from the Sun Yat-Sen University, School of Public Health Ethics Committee.

### Water analysis

Samples were taken from Hengshihe River, irrigating water and wells in the three villages, respectively. Sampling, preservation and analytical protocols were conducted using standard methods for analyzing surface waters as previously described [8,9]. The acid-available fractions of toxic metals (Cd, Cu, Pb, and Zn) were determined. The data quality was controlled by careful standardization, procedural blank measurements, spiked and duplicate samples [8]. The criteria for heavy-metals content were obtained from the Quality Standard for Irrigation Water (GB 5084-1992) and Quality Standard for Ground Water (GB/T 14848-1993), issued by Standardization Administration of People's Republic of China .

### Crops analysis

Rice and avena nula were the major crop species in this geographical region. At each of the three villages, samples were randomly collected in the crop fields. All the crop samples were oven-dried to constant weights. Then, the crop samples were ground by a stainless grinder (Analysis grinder A10, IKA, Germany). Metal concentrations were analyzed by Perkin-Elmer ELAN 6000, an inductively coupled plasma mass spectrometer (ICP-MS). Complete dissolution of samples was performed by the acid digestion method as described by Hernandez et al [10].

### Hair analysis

Hair specimens were obtained from the school-aged children, with either parents or guardians giving their informed consent. The hair sample for each participant was cut into approximately 0.5 cm pieces and then mixed. Each sample was washed with the dilute Triton X-100, and then rinsed with distilled water, de-ionized water and acetone successively [11-13]. The samples were dried in an oven at 80 ± 5°C.

After cutting, each specimen was weighed accurately ( ± 1 mg), then carefully transferred into a 50 ml acid-washed polypropylene centrifuge tube with the addition of ultra-pure trace-metal grade HNO3. The specimen tubes were then introduced into a CEM Mars 5 Plus microwave digestion apparatus. Under the microprocessor control, the hair specimen was subject to the HNO3 acid and a uniform high-temperature digestion via a two-stage temperature ramping sequence. In the first stage, the samples were heated to 70°C and held for 20 min. Then, the temperature was slowly increased (ramped) to 115°C and held for an additional 15 min.

After digestion, the samples were diluted to a set volume with the de-ionized water, recapped and placed on a vortexer for thorough mixing of each hydrated specimen. All the atomic absorption measurements were obtained by using the Polarized Zeeman Atomic Absorption Spectrometry (HITACHI Z-5000, Japan). The accuracy of the results was checked by using a hair reference sample (Chinese reference hair GBW 09191, Shanghai, China). The precision of the atomic absorption measurements was in the range ± 3–5%.

### Assessment of children's behavioural issues

The presences of emotional and behavioural problems of participants were assessed using the Child Behaviour Checklist (CBCL) [14,15], which had been validated in Chinese [16]. The questionnaires were completed by their mothers. Each item in CBCL was scored as 0 (if the problem is not true), 1 (if the problem is somewhat or sometimes true), or 2 (if the problem is very true or often true). The questionnaires (113 items) consisted of eight specific syndrome scales and two broad problem areas, Internalizing and Externalizing, respectively. The Internalizing scale consisted of the syndrome scales (Withdrawn, Somatic Complaints and Anxious/Depressed), whereas the Externalizing scale consists of Delinquent Behaviour and Aggressive Behaviour. The remaining syndrome scales of Social Problems, Thought Problems, and Attention Problems belong neither to the Internalizing nor the Externalizing group. A Total Problems score was obtained by summing the scores over all individual problem items. A higher score indicated a higher level of the problems.

### Statistical analyses

The metal concentrations in different types of samples were compared with the government standards of China. The significant covariates influencing the CBCL subscales of the children were screened by univariate analyses of a list of demographic, geographic and social-economic factors, using the ANOVA F-test or the contingency table chi-square test. Multiple linear regression was then used to evaluate the contribution of hair-metals concentration to each CBCL subscale, after adjusting for socio-demographic factors. Following the typical practice in the studies of childhood metal exposure, hair metals concentration was log transformed (natural log) to remedy its highly skewed distribution. In each case, socio-demographic variables (child's sex, age, towns, and parents' education) were modeled together in the first step. Then the full model including both covariates and the log-transformed hair heavy metals (lead, cadmium, zinc) were constructed. Comparison between the full model and the reduced covariates-only model allowed us to estimate the contribution of combined hair metals to CBCL subscales. Statistical t-test was used to test the regression slope to determine the significance of individual hair metals.

## Results

### Environmental pollution

The downstream part of Hengshihe, the main water source for drinking and irrigation, was seriously polluted by the excretion water from Dabaoshan Mine. The chemical properties of the excretion water, irrigating water and well water in the three studied sites were tested respectively [see Additional file 1]. The excretion water was acidic (PH = 3.35) and the concentration of cadmium and zinc in excretion water reached 7.09×10-3 mg/L and 13.70 mg/L, respectively, being overly higher than the government standards, which is 5.00×10-3 and 1.00 mg/L, respectively. Significantly higher content of cadmium and zinc than the government standards were also found in the irrigation water and wells in Shangba Village, which is closet to Dabaoshan Mine. However, the contents of heavy metals in the irrigation water and wells in other two villages were largely within the government standards [see Additional file 1].

In Shangba and Xiaozhen villages, cadmium and lead concentrations in rice and avena nula were very high. The highest concentrations of cadmium and lead were 0.47 mg/kg (Shangba), and 830 mg/kg (Shangba) in rice and 0.13 mg/kg (Shangba), and 1690 mg/kg (Xiaozhen) in avena nula, respectively [see Additional file 2].

### Social-demographic characteristics and metal level in hair

Of the 549 students with scalp hair samples collected, 245 (44.6%) were males and 304 (55.4%) were females. Among the subjects, 32.6%, 36.1% and 31.3% were from Xiaozhen, Shangba and Dongfang, respectively. The overall mean age was 12.37 (7.17–16.67) years. There was no significant difference of age among subjects from three villages (p > 0.05). The averaged education period for fathers and mothers was 8.2 years and 7.2 years, respectively [see Additional file 3].

The levels of hair lead, cadmium and zinc levels were not normally distributed (median values were 29.32 ug/g, 7.33 ug/g and 61.24 ug/g, respectively). There were weak correlations among hair lead, cadmium and zinc levels (rPb-Cd = 0.249, p < 0.01; rPb-Zn = 0.196, p < 0.01). The median values of hair lead and zinc in Shangba village were significant higher (p < 0.05) than those in other villages [see Additional file 3].

### Emotional and behavioural development score

**Figure 2:**
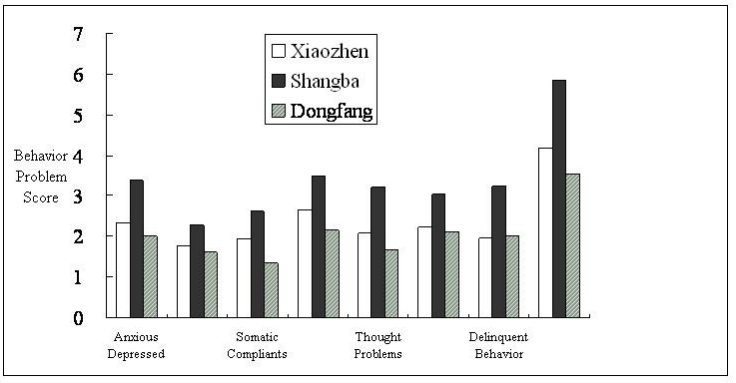
**Averaged Child Behavior Checklist scores for school-aged children in three villages around Dabaoshan mine**. Note that the numbers of items comprising the scales vary, e.g., aggressive behavior consists of 20 behavior problems while thought problems consist of only seven behavior problems.

Total emotional and behavioural development score of the children involved in this study (mean ± standard deviation) was 21.16 ± 18.51. Figure 2 shows the bar plots for the emotional and behavioural development subscale scores of the school-aged children in the three villages. Children from Shangba village had significantly higher scores of total and subscales of behavioural problems than those from other villages (p < 0.01).

The unstandardized multiple regression included all potential confounders (sex, age, family incoming, farther education and mother education) for all CBCL subscale. The set of confounders explained a significant portion of the total variance (range, 0.06–0.10) on each subscale. Boys had significantly more delinquent behaviour problems than girls. Children with higher fathers' education levels had significantly fewer problems on seven subscales, except for somatic complaints, than those with lowers. Children with higher mothers' education levels had significantly fewer problems of anxiety/depression and aggressive behaviours than those with lowers [see Additional file 4 and Additional file 5].

### Relationship between heavy metals exposure and behavioural development

After adjusting for all potential confounders, log-transformed hair lead, cadmium and zinc levels accounted for an incremental of 8% to 15% variance in anxious/depressed, withdrawn, somatic complaints, social problems, thought problems, attention problems, delinquent behaviour and aggressive behaviour. For all subscales, the higher concurrent log-transformed hair lead and zinc exposure levels increase behavioral problems (p < 0.05). But, concurrent log-transformed hair cadmium was only significantly (p < 0.05) associated with withdrawn and social problems [see Additional file 6 and Additional file 7].

## Discussion

Mining activity was a chief source of metals entering into the environment. In the process of mining exploitation and ore concentrating, mine tailing and waste waters were created, and dust was emitted. These industrial endeavours brought tremendous pollutants to the surrounding environment. Since 1970, there had been 35 reported major mine tailing dam failures around the world resulting in significant soil and river pollution and the loss of more than 500 lives [17]. In 2000 alone, there were a total of five reported accidents (in China, Romania, Sweden, and USA) [17].

### Environmental pollution brought by Dabaoshan mine

The Dabaoshan Mining in Guangdong province, China, has been operated for 50 years. The mine tailing dam of this mine collapsed due to heavy rain in 1970. Highly poisonous heavy metals spilled into the Hengshihe River. During this incident, a wide strip of farmland on both sides of the Hengshihe River was covered with a black sludge layer. Although the toxic sludge and a major portion of the contaminated soil surface were mechanically removed, most of the contaminated farmlands and waters are continuously polluted with the spills from the mine. Unfortunately, a part of these contaminated farmlands and waters are still used by local residents today.

This cross-sectional study was carried out before the rainy season (September to October) in Hengshihe River area in 2006. The spilled mine tailing and the concentrations of heavy metal in the studied sites may be much less than what would be measured in a rainy season. Even though, compared with the government standards in China, the heavy-metals pollution in this area were actually over the thresholds. For example, the PH value of the water in the Hengshihe River across Shangba Village was highly acidic, with a value of 4.92. Furthermore, the soils in the vast downstream area, as a long-term sink for potentially toxic elements, were increasingly polluted by more and more depositing heavy metals, including lead, cadmium, copper and zinc. In Shangba and Xiaozhen villages where the Hengshihe River runs through, the crops such as rice were polluted by uptaking heavy metals through their roots from the contaminated soils. The local residents in turn absorbed heavy metals by eating the rice grown in this region. Compared with the government standard, the concentrations of heavy metals in the rice grown in these villages were higher than the critical maximum levels of heavy metals in rice, established to protect the residents' health [18].

### Children's behavioural development

The high concentration of heavy metals in water, soil and crops, is imposing a strong threat on the health of the local residents. Continuous uptake of heavy metals has cumulative effects on the residents, especially for children, since there is not an efficient way for elimination of the heavy-metals residue in child bodies, and the detrimental impact, either physiological or mental, can become apparent only after several yeas of exposure [19,20].

The emotional and behavioural problems survey showed that the unadjusted levels of behaviour problems differed across the study sites, with Shangba Village being at the highest level. According to fact that heavy metal pollution in Shangba is the most severe, which may explain in part of the highest prevalence of behaviour problems in this village? In our study, one of the influential factors on the eight subscale scores of the emotional and behavioural problems is the parents' education, indicating that the parents' ignorance of the detriments brought by heavy metals may increase the environmental exposure to their children, thus leading to the higher occurrence of heavy-metals-induced psychological problems in their offspring.

### Relationship between heavy metal concentration and behavioural development

The present study suggests that there is a significant relation between measured lead concentration and scores on the Child Behaviour Checklist. Measured hair lead is significantly associated with scores on the eight specific behaviour syndrome scales for school children aged 7–16 years.

Although the cross-sectional design of the present study cannot produce a causal association between lead and children's behavioural problems, it does provide evidence for the hypothesis that lead exposure has long term effects on the prevalence of behavioural and emotional problems. There are a number of arguments in favour of this hypothesis. First, the neurotoxic effects of exposure to high levels of lead are well known and documented [21-25], and case reports have suggested a causal link between lead exposure and psychiatric status [26-28]. Second, animal experiments that have examined the effect of lead exposure have found adverse effects on both early mother-infant interaction and social play, and have reported increased aggression and hyperactivity in exposed offspring [29].

Consistent with the support for the impact of lead on behaviour problems, the magnitude of lead effect is very large. After adjusting for confounders (sex, age, family incoming, farther education and mother education), measured heavy metals, especially lead, account for about 10% to 15% of the variance in the subscale scores. Slight disturbances in early behaviour, coupled with the known adverse effect of lead on intelligence [30,31], may compound over time, contributing to more substantial difficulties by adult age. It is important to strengthen the management of environmental pollution conditions in the Hengshihe area, and to improve the children's health through lowering their body lead concentration.

This study was hindered by some unavoidable limitations. Of particular importance is the limited information about potential confounders. Due to the limited financial support and human resource, our study aimed to survey the pollution condition and children' health status during our study period, and then estimated their preliminary relationship in the Dabaoshan area. More comprehensive information about potential confounders would be obtained by further study.

## Conclusion

In conclusion, heavy metals including lead, cadmium and zinc were accumulated in water and crops in Hengshihe River pollution area and other conclusions, such as the effects of heavy metal exposures on behavioural problems. Given the increasing number of children at risk for lead and cadmium exposure with advent of industrialization especially in developing countries like China, the control and prevention strategies for abating toxic heavy-metal exposure and appropriate treatment on a variety of heavy-metals-induced psychological behavioural problems deserve serious attention among both epidemiologists and clinical physicians.

## Competing interests

The authors declare that they have no competing interests.

## Authors' contributions

QSB and CYL conceived the study, and participated in its design and helped to draft the manuscript. HS, MW, WHL, WQC and XQD collected the data and helped to draft the manuscript. YTH and SQR performed the statistical analysis and drafted the manuscript. All authors read and approved the final manuscript.

## Pre-publication history

The pre-publication history for this paper can be accessed here:



## Supplementary Material

Additional file 1 **Heavy metals concentration in water source, irrigating water and well around a multi-metals sulfide mine in Guangdong, China**. The data provided the heavy metals concentration in water source, irrigating water and well of this study. Click here for file

Additional file 2 **Heavy metals concentration in soil, rice and avena nula around a multi-metals sulfide mine in Guangdong, China**. The data provided the heavy metals concentration in soil, rice and avena nula of this study. Click here for file

Additional file 3 **Sample characteristics of school-aged children living around a multi-metals sulfide mine in Guangdong, China**. The table described the sample characteristics, including sex, age, father education, mother education, hair heavy metal concentration and CBCL total score. Click here for file

Additional file 4 **Effect of socio-demographic factors on the Child Behavior Checklist Subscale score of school-aged children living around a mine, Guangdong, China**. The table showed the effect of socio-demographic factors on four CBCL subscale scores (Anxious/Depressed, Withdrawn, Somatic Complaints, Social Problems). Click here for file

Additional file 5 **Effects of socio-demographic factors on the Child Behavior Checklist Subscale Score of school-aged children in Mining area, Guangdong, China**. The table showed the effect of socio-demographic factors on the other four CBCL subscale scores (Thought Problems, Attention Problems, Delinquent Behavior, Aggressive Behaviors). Click here for file

Additional file 6 **Effects of hair heavy metals concentration on the Child Behavior Checklist Subscale scores of school-aged children in the mining area, Guangdong, China**. The table showed the effect of hair heavy metals concentration on four CBCL subscale scores (Anxious/Depressed, Withdrawn, Somatic Complaints, Social Problems). Click here for file

Additional file 7 **Effects of hair heavy metals concentration on the Child Behavior Checklist Subscale scores of school-aged children in the mining area, Guangdong, China**. The table showed the effect of hair heavy metals concentration on the other four CBCL subscale scores (Thought Problems, Attention Problems, Delinquent Behavior, Aggressive Behaviors). Click here for file
