# Supplementary material for: Behavioural development of school-aged children who live around a multi-metal sulphide mine in Guangdong province, China: a cross-sectional study
Source: BMC Public Health. 2009 Jul 3;9:217. doi: 10.1186/1471-2458-9-217 (PMC2717083; doi:10.1186/1471-2458-9-217)
Supplement: Additional file 1 — Heavy metals concentration in water source, irrigating water and well around a multi-metals sulfide mine in Guangdong, China. The data provided the heavy metals concentration in water source, irrigating water and well of this study. [file 1471-2458-9-217-S1.doc]

## Table 1 - Heavy metals concentration in water source, irrigating water and well around a multi-metals sulfide mine in Guangdong, China

|  | Water source (mg/L) | | Irrigating water (mg/L) | | | | Well (mg/L) | | | |
| --- | --- | --- | --- | --- | --- | --- | --- | --- | --- | --- |
|  | Excretion water | GS | Shangba | Xiaozhen | Dongfang | GS | Shangba | Xiaozhen | Dongfang | GS |
| PH | 3.35 | 6.0-9.0 | 4.92 | 7.75 | 7.76 | 5.50-8.50 | 4.77 | 7.06 | 6.88 | 6.5-8.5 |
| Cadmium | 7.09×10^-3^ | 5×10^-3^ | 8.31×10^-3^ | 4.25×10^-3^ | 0.66×10^-3^ | 5×10^-3^ | 8.88×10^-3^ | 0.13×10^-3^ | 0.07×10^-3^ | 5.0×10^-3^ |
| Lead | 0.043 | 0.010 | 0.045 | 0.035 | 0.013 | 0.200 | 0.012 | 0.017 | 0.016 | 0.01 |
| Copper | 0.196 | 1.000 | 0.631 | 0.020 | 0.020 | 0.500 | 1.570 | 0.020 | 0.045 | 1.00 |
| Zinc | 13.700 | 1.000 | 25.200 | 0.048 | 0.005 | 2.000 | 4.89 | 0.024 | 0.011 | 1.00 |

Note: 1. GS: government standard.

2. – no government standard
