# Supplementary material for: Behavioural development of school-aged children who live around a multi-metal sulphide mine in Guangdong province, China: a cross-sectional study
Source: BMC Public Health. 2009 Jul 3;9:217. doi: 10.1186/1471-2458-9-217 (PMC2717083; doi:10.1186/1471-2458-9-217)
Supplement: Additional file 2 — Heavy metals concentration in soil, rice and avena nula around a multi-metals sulfide mine in Guangdong, China. The data provided the heavy metals concentration in soil, rice and avena nula of this study. [file 1471-2458-9-217-S2.doc]

## Table 2 - Heavy metals concentration in soil, rice and avena nula around a multi-metals sulfide mine in Guangdong, China

|  | Soil (mg/kg) | | | | Rice (mg/kg) | | | | Avena Nula (mg/kg) | | | |
| --- | --- | --- | --- | --- | --- | --- | --- | --- | --- | --- | --- | --- |
|  | Shangba | Xiaozhen | Dongfang | GS | Shangba | Xiaozhen | Dongfang | GS | Shangba | Xiaozhen | Dongfang | GS |
| Cadmium | 0.528 | 0.422 | 0.043 | 0.30 | 0.47 | 0.36 | 0.02 | 0.20 | 0.13 | 0.06 | 0.01 | 0.20 |
| Lead | 600 | 770 | 68 | 300 | 830 | 520 | 280 | 300 | 820 | 1690 | 1050 | 300 |
| Copper | 1261 | 147 | 20 | 100 | 5.38 | 3.08 | 2.03 | 10 | 1.31 | 0.82 | 0.42 | - |
| Zinc | 680 | 227 | 68 | 250 | 29.40 | 20.70 | 13.80 | 50 | 20.80 | 5.12 | 3.73 | - |

Note: 1. GS: government standard.

2. – no government standard
