# Supplementary material for: Behavioural development of school-aged children who live around a multi-metal sulphide mine in Guangdong province, China: a cross-sectional study
Source: BMC Public Health. 2009 Jul 3;9:217. doi: 10.1186/1471-2458-9-217 (PMC2717083; doi:10.1186/1471-2458-9-217)
Supplement: Additional file 3 — Sample characteristics of school-aged children living around a multi-metals sulfide mine in Guangdong, China. The table described the sample characteristics, including sex, age, father education, mother education, hair heavy metal concentration and CBCL total score. [file 1471-2458-9-217-S3.doc]

## Table 3 - Sample characteristics of school-aged children living around a multi-metals sulfide mine in Guangdong, China

|  | | Xiaozhen  (n=179) | Shangba  (n=198) | Dongfang  (n=172) | Total  (n=549) | *p* value |
| --- | --- | --- | --- | --- | --- | --- |
| Sex | |  |  |  |  |  |
|  | Male | 74 (41.3%) | 95 (48.0%) | 76 (44.2%) | 245 (44.6%) | 0.428 ^c^ |
|  | Female | 105 (58.7%) | 103 (52.1%) | 122 (55.8%) | 304 (55.4%) |  |
| Age, y | | 12.18 (2.41) | 12.32 (2.14) | 12.61 (2.05) | 12.37 (2.21) | 0.177^d^ |
| Father education, y | | 8.56 (2.53) | 7.89 (2.94) | 8.19 (2.97) | 8.20 (2.83) | 0.071 ^d^ |
| Mother education, y | | 7.33 (2.44) | 7.26 (2.60) | 7.05 (2.48) | 7.22 (2.51) | 0.563 ^d^ |
| Hair Lead, ^a^ μg/g | | 3.54 (0.10-58.24) | 5.35 (0.48-24.70) | 3.06 (0.08-31.17) | 4.19 (0.08-58.24) | <0.001^e^ |
| Hair Cadmium, ^a^μg/g | | 0.12 (0.01-6.85) | 0.10 (0.04-2.14) | 0.09 (0.01-1.01) | 0.10 (0.01-6.85) | 0.026 ^e^ |
| Hair Zinc, ^a^ μg/g | | 173.45 (65.16-884.61) | 262.37 (32.71-1295.99) | 204.60 (76.97-517.80) | 211.52 (32.71-1295.99) | <0.001 ^e^ |
| CBCL ^b^ total score (SD) | | 56.25 (11.81) | 61.19 (10.82) | 54.54 (10.83) | 57.50 (11.50) | <0.001 ^d^ |

*Note*. Dongfang is the non-exposed village.

^a^Concentration of heavy metals was showed as media value (range).

^b^Child Behavior Checklist.

^c^Difference between three sites is assessed by chi-square test.

^d^Difference between three sites is assessed by ANOVA analysis.

^e^Difference between three sites is assessed by a nonparametric test.
