# Supplementary material for: Behavioural development of school-aged children who live around a multi-metal sulphide mine in Guangdong province, China: a cross-sectional study
Source: BMC Public Health. 2009 Jul 3;9:217. doi: 10.1186/1471-2458-9-217 (PMC2717083; doi:10.1186/1471-2458-9-217)
Supplement: Additional file 4 — Effect of socio-demographic factors on the Child Behavior Checklist Subscale score of school-aged children living around a mine, Guangdong, China. The table showed the effect of socio-demographic factors on four CBCL subscale scores (Anxious/Depressed, Withdrawn, Somatic Complaints, Social Problems). [file 1471-2458-9-217-S4.doc]

## Table 4 - Effect of socio-demographic factors on the Child Behavior Checklist Subscale score of school-aged children living around a mine, Guangdong, China

|  | Anxious/Depressed | | | Withdrawn | | | Somatic Complaints | | | Social Problems | | |
| --- | --- | --- | --- | --- | --- | --- | --- | --- | --- | --- | --- | --- |
|  | B^a^ | SE | *p* | B^a^ | SE | *p* | B^a^ | SE | *p* | B^a^ | SE | *p* |
| Sex ^b^ | 0.190 | 0.252 | 0.452 | 0.109 | 0.178 | 0.543 | 0.292 | 0.230 | 0.205 | -0.219 | 0.223 | 0.327 |
| Age | 0.217 | 0.057 | 0.000 | 0.175 | 0.041 | 0.000 | 0.249 | 0.052 | 0.000 | 0.056 | 0.051 | 0.271 |
| Family incoming | -0.246 | 0.115 | 0.032 | -0.222 | 0.081 | 0.007 | -0.356 | 0.105 | 0.001 | -0.252 | 0.102 | 0.013 |
| Farther Education | -0.147 | 0.046 | 0.001 | -0.096 | 0.032 | 0.003 | -0.089 | 0.042 | 0.033 | -0.163 | 0.040 | 0.000 |
| Mother Education | -0.101 | 0.052 | 0.050 | -0.032 | 0.036 | 0.380 | -0.032 | 0.047 | 0.499 | -0.062 | 0.046 | 0.172 |
| Overall R^2^ | 0.07 | | | 0.06 | | | 0.06 | | | 0.06 | | |

^a^estimated regression coefficient.

^b^boys=0; girls=1.
