# Supplementary material for: Behavioural development of school-aged children who live around a multi-metal sulphide mine in Guangdong province, China: a cross-sectional study
Source: BMC Public Health. 2009 Jul 3;9:217. doi: 10.1186/1471-2458-9-217 (PMC2717083; doi:10.1186/1471-2458-9-217)
Supplement: Additional file 5 — Effects of socio-demographic factors on the Child Behavior Checklist Subscale Score of school-aged children in Mining area, Guangdong, China. The table showed the effect of socio-demographic factors on the other four CBCL subscale scores (Thought Problems, Attention Problems, Delinquent Behavior, Aggressive Behaviors). [file 1471-2458-9-217-S5.doc]

## Table 5 - Effects of socio-demographic factors on the Child Behavior Checklist Subscale Score of school-aged children in Mining area, Guangdong, China.

|  | Thought Problems | | | Attention Problems | | | Delinquent Behavior | | | Aggressive Behavior | | |
| --- | --- | --- | --- | --- | --- | --- | --- | --- | --- | --- | --- | --- |
|  | B^a^ | SE | *p* | B^a^ | SE | *p* | B^a^ | SE | *p* | B^a^ | SE | *p* |
| Sex ^b^ | -0.025 | 0.233 | 0.916 | -0.379 | 0.196 | 0.053 | -0.902 | 0.211 | 0.000 | -0.472 | 0.361 | 0.192 |
| Age | 0.113 | 0.053 | 0.034 | 0.036 | 0.045 | 0.414 | 0.008 | 0.048 | 0.861 | -0.028 | 0.082 | 0.733 |
| Family incoming | -0.367 | 0.106 | 0.001 | -0.205 | 0.089 | 0.022 | -0.159 | 0.096 | 0.099 | -0.246 | 0.164 | 0.135 |
| Farther Education | -0.160 | 0.042 | 0.000 | -0.154 | 0.035 | 0.000 | -0.176 | 0.038 | 0.000 | -0.186 | 0.065 | 0.005 |
| Mother Education | -0.035 | 0.048 | 0.463 | -0.051 | 0.040 | 0.200 | -0.057 | 0.043 | 0.186 | -0.137 | 0.074 | 0.063 |
| Overall R^2^ | 0.06 | | | 0.10 | | | 0.09 | | | 0.04 | | |

^a^estimated regression coefficient.

^b^boys=0; girls=1.
