# Supplementary material for: Behavioural development of school-aged children who live around a multi-metal sulphide mine in Guangdong province, China: a cross-sectional study
Source: BMC Public Health. 2009 Jul 3;9:217. doi: 10.1186/1471-2458-9-217 (PMC2717083; doi:10.1186/1471-2458-9-217)
Supplement: Additional file 6 — Effects of hair heavy metals concentration on the Child Behavior Checklist Subscale scores of school-aged children in the mining area, Guangdong, China. The table showed the effect of hair heavy metals concentration on four CBCL subscale scores (Anxious/Depressed, Withdrawn, Somatic Complaints, Social Problems). [file 1471-2458-9-217-S6.doc]

## Table 6 - Effects of hair heavy metals concentration on the Child Behavior Checklist Subscale scores of school-aged children in the mining area, Guangdong, China

|  | Anxious/Depressed | | | Withdrawn | | | Somatic Complaints | | | Social Problems | | |
| --- | --- | --- | --- | --- | --- | --- | --- | --- | --- | --- | --- | --- |
|  | B^a^ | SE | *p* | B^a^ | SE | *p* | B^a^ | SE | *p* | B^a^ | SE | *p* |
| Hair Lead, μg/g | 0.894 | 0.111 | <0.001 | 0.410 | 0.081 | 0.000 | 0.743 | 0.105 | 0.000 | 0.676 | 0.099 | .000 |
| Hair Cadmium, μg/g | 0.227 | 0.121 | 0.061 | 0.282 | 0.088 | 0.001 | 0.030 | 0.114 | 0.793 | 0.274 | 0.108 | .012 |
| Hair Zinc, μg/g | -1.170 | 0.212 | 0.000 | -0.792 | 0.154 | 0.000 | -0.709 | 0.200 | 0.000 | -1.053 | 0.190 | .000 |
| △R^2^ | 0.15 | | | 0.11 | | | 0.11 | | | 0.14 | | |

*Note.* The change in R^2^ represents the proportion of variance account for by hair lead, cadmium and zinc, after control for the variables in the full model. Each △R^2^ is calculated as the difference in total model R^2^, with and without the hair heavy metals measures.

^a^Estimated regression coefficient for log hair lead, cadmium and zinc, adjusted for sex, age, family incoming, farther education and mother education.
