# Supplementary material for: Behavioural development of school-aged children who live around a multi-metal sulphide mine in Guangdong province, China: a cross-sectional study
Source: BMC Public Health. 2009 Jul 3;9:217. doi: 10.1186/1471-2458-9-217 (PMC2717083; doi:10.1186/1471-2458-9-217)
Supplement: Additional file 7 — Effects of hair heavy metals concentration on the Child Behavior Checklist Subscale scores of school-aged children in the mining area, Guangdong, China. The table showed the effect of hair heavy metals concentration on the other four CBCL subscale scores (Thought Problems, Attention Problems, Delinquent Behavior, Aggressive Behaviors). [file 1471-2458-9-217-S7.doc]

## Table 7 - Effects of hair heavy metals concentration on the Child Behavior Checklist Subscale scores of school-aged children in the mining area, Guangdong, China

|  | Thought Problems | | | Attention Problems | | | Delinquent Behavior | | | Aggressive Behavior | | |
| --- | --- | --- | --- | --- | --- | --- | --- | --- | --- | --- | --- | --- |
|  | B^a^ | SE | *p* | B^a^ | SE | *p* | B^a^ | SE | *p* | B^a^ | SE | *p* |
| Hair Lead, μg/g | 0.856 | 0.104 | 0.000 | 0.606 | 0.089 | 0.000 | 0.752 | 0.095 | 0.000 | 1.207 | 0.162 | 0.000 |
| Hair Cadmium, μg/g | 0.201 | 0.113 | 0.077 | 0.194 | 0.097 | 0.045 | 0.102 | 0.104 | 0.324 | 0.216 | 0.176 | 0.222 |
| Hair Zinc, μg/g | -0.729 | 0.198 | 0.000 | -0.660 | 0.169 | 0.000 | -0.641 | 0.181 | 0.000 | -1.420 | 0.309 | 0.000 |
| △R^2^ | 0.15 | | | 0.08 | | | 0.12 | | | 0.13 | | |

*Note.* The change in R^2^ represents the proportion of variance account for by hair lead, cadmium and zinc, after control for the variables in the full model. Each △R^2^ is calculated as the difference in total model R^2^, with and without the hair heavy metals measures.

^a^Estimated regression coefficient for log hair lead, cadmium and zinc, adjusted for sex, age, family incoming, farther education and mother education.
